# Cysto-Urethroscopic Observations of Post-Gonorrheal Conditions in the Prostatic Urethra and Bladder Neck Region

**Published:** 1919

**Authors:** 


***Cysto-Urethroscopic Observations of Post-Gonorrheal Conditions in the Prostatic Urethra and Bladder Neck Region.*** By Capt. CLINTON K. SMITH. *Urologic and Cutaneous Review*, October, 1918.

One hundred cases were examined from the " general run of patients designated as 'chronic gonorrhea' " at the Isolation Section for Venereal Diseases at Kelly Field.

Examination of patients on admission showed : —

Average time since the incidence of the infection 27.84 months.

Gonococci in urethral secretions in 24 cases. Urine one and two cloudy in 24 cases ; one cloudy and two clear in 29 cases ; one and two clear in 47 cases.

Prostate gland and seminal vesicles tender to rectal palpation in 94 and 95 cases respectively.

Expressed secretions from prostate gland and seminal vesicles contained pus in all cases, gonococci in 16 cases.

A cysto-urethroscopic examination was made soon after treatment was begun in cases where there were no gonococci in the urethral secretions ; in the other cases examination was not made till the infection was eliminated, on an average, 15 to 20 days.

At the time of this examination, all urethral secretions contained pus.

In the bladder, the trigone was congested and abnormally red, especially toward the apex in 36 cases.

In prostatic urethra, verumontanum was abnormally large, highly congested, bleeding easily in 51 cases.

Prostatic ducts visible in 37 cases. Pus was seen exuding from these ducts in 15 cases. Pressure on the prostate per rectum increased the amount of pus.

Ejaculatory ducts identified in three cases; pus from these ducts seen in 2 cases.

Walls of the prostatic urethra congested and granular in 19 cases.

Membrane of the anterior urethra normal in most cases ; slight granulations in a small percentage. A. F. 24 cystoscope passed without difficulty in all cases.

Treatment directed toward clearing the urethra of gonococci ; toward eliminating the infection from prostate and seminal vesicles; toward correcting pathological changes in these structures. Treatment not described in detail ; author mentions that where pus was seen exuding from prostatic ducts, vigorous prostatic massage was part of the treatment. In some cases vasotomy and vaso-puncture with vesicle installation with an argyrol solution were done.

The author's conclusions are : —

A protest is due against the use of the term " chronic gonorrhea " without qualifications. It is unscientific. It encourages a viewpoint that is incompatible with the proper treatment of gonorrheal infection involving the prostate gland, seminal vesicles and allied structures.

The evidence submitted suggests the almost universal involvement of the prostate and seminal vesicles or their urethral exits, in those cases in which the gonococcus establishes a habitat back of the cut-off muscle.

In order to properly treat these cases one should be able to specify, with a reasonable degree of certainty, the lesion and the pathology which he is attempting to correct.

The cysto-urethroscopic observations revealed in the high percentage of the cases an inflammatory condition of the urethral exits of the prostate and seminal vesicles. In the majority of the cases, as no evidence could be obtained to demonstrate the presence of the gonococcus, it seemed logical to designate the condition as post-gonorrheal.

The cysto-urethroscopic examinations were of assistance in establishing the conviction that the pus which was persistently present in the urethral secretions, had its origin, in most instances, in the prostate gland, the seminal vesicles, or the ducts and orifices of these structures.

The disappearance of the pus following treatment directed towards the correction of pathological changes in the prostate, seminal vesicles, and allied structures, largely substantiated this conclusion.

